# Binding Energy Calculations of Anthracene and Rhodamine 6G H-Type Dimers: A Comparative Study of DFT and SMD Methods

**DOI:** 10.1021/acs.jpca.4c07867

**Published:** 2025-02-06

**Authors:** Daniel Doveiko, Karina Kubiak-Ossowska, Yu Chen

**Affiliations:** 1 Photophysics Group, Department of Physics, Scottish Universities Physics Alliance, University of Strathclyde, Glasgow G4 0NG, U.K.; 2 Department of Physics/Archie-West HPC, University of Strathclyde, 107 Rottenrow, Glasgow G4 0NG, U.K.

## Abstract

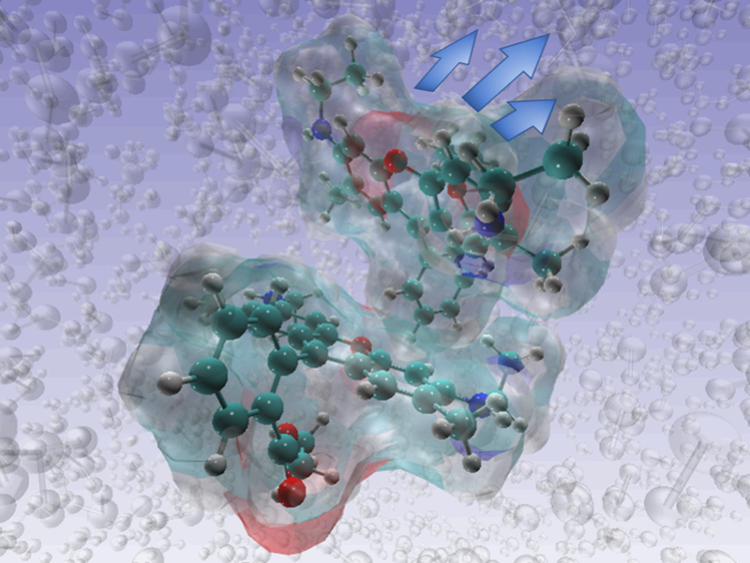

With the ever-growing need to study systems of increased size and complexity, modern density functional theory (DFT) methods often encounter problems arising from the growing computational demands. In this work, we have presented a comprehensive DFT validation of the steered molecular dynamics (SMD) approach in estimating the binding energies of aromatic dimers. By performing DFT calculations on optimized and unoptimized anthracene and rhodamine 6G (R6G) dimers using functionals with progressively enhanced exchange-correlation energy description and comparing the obtained results with SMD-predicted values, it was found that SMD predictions are in good agreement with the results obtained from hybrid DFT calculations. The average binding energies for optimized anthracene dimers were found to be 6.46 kcal/mol using DFT at ωB97X-D4/def2-QZVPP and 7.64 ± 1.61 kcal/mol as predicted by the SMD. For the R6G H-type dimer, the binding energies were 17.48 and 19.02 ± 2.22 kcal/mol, respectively. The study also revealed that due to the lack of explicit terms accounting for electron–electron interactions in MD force fields, the proposed method tends to overbind dimers. It is anticipated that the presented method can be applied to more complex dimers, potentially accelerating the calculations of binding energies. Moreover, this study further validates the accuracy of the CHARMM36 FF.

## Introduction

In recent decades, Kohn–Sham Density Functional Theory (KS-DFT)^1^ has become a primary tool to probe the electronic structure of molecules and address many-electron problems, which are crucial for advancing our understanding of modern physics, chemistry, and biology.^2,3^

The popularity of DFT stems from its excellent balance between its reliability and computational cost, offering significantly greater accuracy than modern semiempirical methods while being much less computationally demanding than the gold standard of the field: the Coupled Cluster with Singles, Doubles, and Perturbative Triples (CCSD(T)) approach.^4,5^ However, the CCSD(T) model,^6^ scales as N^7^ (where N is a measure of the system size), requiring a substantial amount of computational resources. Other sophisticated post-Hartree–Fock (HF) methods such as the second-order many-body perturbation theory with the Mo̷ller–Plesset partitioning of the Hamiltonian (MP2)^7^ scales as N^5^. While more feasible than CCSD(T), MP2 is still a rather demanding approach. Efforts to speed up those methods, such as the domain-based local pair-natural orbital (DLPNO) methods, resulting in DLPNO-CCSD(T) with nearly linearly scaling, or the RIJCOSX-MP2^8^ method, have been developed. However, the requirement for a large basis set in post-HF methods, needed to minimize the basis set superposition error (BSSE), limits the system size that can be effectively calculated using those approaches.^9,10^

On the other hand, the theoretical details and caveats of DFT are very well understood, with ongoing improvements addressing its weakness, such as the addition of dispersion correction by Grimme’s group,^11−13^ which mitigates known significant drawbacks of DFT. Furthermore, DFT is considered a robust theory, and failures in the form of completely erroneous results are relatively rare, even when applied to complex molecules or an exotic system. This reliability has made DFT a black-box-like method that nonexperts can successfully apply to various problems without needing to delve deeply into the complex underlying theory.^14^ However, accurately interpreting DFT results still requires specialist knowledge.

Nonetheless, calculations of large systems are still very demanding, even with the additions mentioned above and different approximations such as “Resolution of the Identity” (RI)^15^ and “Chains of Sphere” (COSX)^16^ as implemented in ORCA^17^ or SENEX^18^ in TURBOMOLE,^19^ and the help of state-of-the-art High-Performance Computers (HPCs). A method that provides faster yet comparable results is essential for accelerating scientific progress. To address this issue, we present a DFT-based validation of a Steered Molecular Dynamics approach (SMD), which we already successfully applied to anthracene and phenanthrene H-type dimers.^20^ In this study, we extend it to investigate Rhodamine 6G (R6G) H-type dimers comprehensively by applying a very systematic and progressive approach and covering functionals of increasing complexity. Compared with our previous work, we perform calculations on a wide range of dimers, each having different conformations for both optimized and unoptimized structures, to explore the effect of diverse geometries on the measured binding energies. This highlights the novelty of employing the SMD approach for accurately assessing the binding energies. The presented method is not intended to replace QM/DFT entirely but to complement them by addressing specific computational challenges. While DFT remains a powerful and widely used tool in modern computational chemistry, the presented SMD method enables significantly faster binding energy assessments of large molecules without compromising the overall framework of DFT in broader applications.

The first Molecular Dynamics (MD) simulations of 32 hard spheres were performed in the late 1950s by Alder and Wainwright^21^ and were based on classical equations of motion, where the evolution of the many-body system was solved numerically.^22^ The interactions within the system were described using force fields (FF), which contained all of the parameters needed to evaluate the complex interactions of the various components studied. Subsequently, with the advances in NMR spectroscopy^23^ and X-ray crystallography,^24^ more molecular structures became available; hence, MD simulations became a well-established and irreplaceable tool for the investigation of biomolecules,^25^ lipids,^26^ and complex inorganic nanocomposites.^27^ With the recent addition of modern graphics processing units (GPUs), such simulations became feasible on a rather long simulation time scales at a modest cost.^28,29^ As a result, MD can also be used to study complex interactions of small biomolecules with inorganic structures^30^ on a large scale, providing atomic-level insights into these systems and significantly simplifying the interpretation of real-world experiments. SMD, a variant of MD, applies external force to the system, enabling the exploration of processes such as the ligand unbinding and molecular conformational changes.^31^ Additionally, SMD has demonstrated excellent agreement with experimental techniques, such as MP-SPR and AFM.^32−34^

Rhodamine 6G (R6G), also known as Rhodamine 590, is a xanthene dye frequently used in dye lasers and as a fluorescent tracer.^35^ It exhibits exceptional photostability in various solvents and in a wide pH range while maintaining a high quantum yield.^36^ Its chemical structure, consisting of a xanthene core with three aromatic rings in a single plane, its hydrophobicity and cationic charge allow it to easily bind to a wide range of compounds, such as silica nanoparticles, sodium silicates,^37,38^ gold nanoparticles,^39,40^ titanium dioxide nanocomposites,^41^ and graphene.^42,43^ Additionally, R6G can be used as a sensor for mercury(II) detection in water^44^ and for the specific and sensitive detection of nitrite.^45^ Given its broad applications, understanding its properties is crucial.

One important feature that directly affects the usability of R6G is its tendency to form dimers at high concentrations. The H-type aggregates that are formed are not fluorescent, so obtaining the accurate values of the dimer binding energies is important for optimizing constructs based on R6G's fluorescent properties. Because of its importance in sensing and due to its stability and aromatic structure, R6G serves as an excellent model for studying dimer stability and binding energy using both DFT and SMD. Measurements of dimerization energies, especially in solutions, are experimentally very demanding, or even currently impossible, so computational estimates of stability of dimers in solutions are irreplaceable.

In this work, the applicability of SMD in calculating binding energies of dimers will be addressed, and the proposed method will be described in detail. Furthermore, the approach is validated using DFT calculations by employing functionals from various rungs of Perdew’s “Jacob’s ladder”, which classifies the functionals based on their accuracy in predicting exchange-correlation energy.^46^ While there are much more sophisticated *ab initio* and force field methods that can potentially report even more reliable binding energies, the drawback of such methods, as mentioned previously, lies in their computational cost and complexity. The main goal of this work was to show that simple methods, such as SMD can successfully report accurate binding energies comparable with DFT results. Furthermore, due to the dimerization mechanism in both anthracene and R6G dimers, which are mainly driven by stacking interactions, the measurement of binding energies experimentally is virtually impossible; hence, the only reasonable methods are based on computations.^47,48^ The comprehensive DFT and SMD calculations presented in this work show that simple force field methods used in this novel and unusual approach can be successfully used to assess the binding energies of aromatic dimers.

## Methods

### Dimer Generation Using MD

As R6G required nonstandard parameters, those were obtained in a multistep method. First, the initial structure of the dye was uploaded into the PDB Reader^49,50^ of CHARMM-GUI,^51^ which generated the initial parameters and topology files using CGenFF.^52^ Next, the obtained parameters were corrected according to those obtained using automated frequency matching and reported by Vaiana et al.,^53^ while the partial charges used were based on the values reported by Chuichay et al.^54^ The R6G parametrization process involves the use of the CHARMM36 force field in all MD and SMD simulations. For the MD simulations used to generate the initial dimer structures, two anthracene or R6G molecules were placed in a water box and allowed to diffuse freely. To ensure that the individual monomers are not biased toward dimerization, the distance between them was ∼20 Å, with the water box padding 20 Å to exclude interactions with the periodic image. The rectangular periodic simulation cell size was 67 Å × 70 Å × 65 Å and contained 27,442 atoms for R6G simulations, while for the anthracene simulations, the cell size was 72 Å × 50 Å × 46 Å (14,781 atoms). Initially, the system underwent 1000-step water-only minimization using the steepest descent method with all nonsolvent molecules restrained followed by 100 ps equilibration at 300 K and 1 bar maintained via the Langevin barostat and thermostat (NPT ensemble). The obtained system was subject to 10,000 minimization steps with no constraints applied, followed by 30 ps of heating to 300 K and 270 ps equilibration with a 1 fs time step. The production runs were performed in the NVT ensemble, where a Langevin thermostat with 5 ps^–1^ damping was used to control the temperature with a 1 fs time step integrator. The 100 ns production run at 300 K was repeated four times to ensure that the system was not in a saddle point and to generate four unique dimers independently. A custom TCL script was used to measure the center of mass (COM) distance between two dye molecules. This, combined with visual analysis, allowed the identification and selection of four unique dimers, one from each simulation that was used as a starting structure for both the SMD simulations and DFT calculations. In all cases, Particle Mesh Ewald (PME) with 1.0 Å grid spacing was used for the fast evaluation of the electrostatic interactions, while the cut off for the vdW interactions was set to 12 Å. A sample MD trajectory and the TCL COM script are provided in the Supporting Information.

### Density Functional Theory Calculations and Geometry Optimization Methods

Four independent dimers were used as starting points for the DFT calculations. All calculations were performed using ORCA 5.0.4.^17,55,56^ To get a valid comparison between the estimated energies using our proposed method and those of conventional DFT, binding energies were calculated at multiple levels of theory as follows:

(1) Generalized gradient approximation (GGA) functional: BP86,^57^ which combines Becke’s exchange functional (B88)^57^ and Perdew’s (P86)^58^ correlation functional and improves on local density approximation by incorporating the gradient of the electron density;
(2) Hybrid GGA: Becke (3 parameter)–Lee–Yang–Parr (B3LYP) functional,^59−61^ which is one the most widely used functionals and includes a portion of exact HF exchange, thus further improving the accuracy of the calculated electronic properties;
(3) Hybrid-meta-GGA: M06-2X,^62^ a global hybrid, which is a high-nonlocality functional and includes both gradient and kinetic energy density;
(4) Range-separated hybrid: ωB97X-D,^63,64^ which separates the exchange interaction into short-range and long-range components by applying different treatments to each.

For BP86, B3LYP, and ωB97X-D functionals, Grimme’s atom-pairwise dispersion correction based on tight binding partial charges (D4)^65−67^ was used, while for the M06-2X functional, D3 dispersion correction with a zero-damping scheme was used^11^ due to a lack of available parameters for the D4 correction. At each step, starting from the lowest (BP86) to the highest level of theory (ωB97X-D), the dimer and two individual monomers were optimized in a conductor-like polarizable continuum solvation model (CPCM, water), followed by harmonic frequency calculations to confirm that the obtained stationary point is a minimum. The zero-point energy (ZPE) and thermal corrections were applied to the obtained energies, while basis set superposition error (BSSE) correction was applied according to Boys and Bernardi procedure,^68^ to account for the basis set incompleteness effect. Triple ζ valence def2-TZVPP basis set^69^ with auxiliary def2/J^70^ was used for all optimization and frequency calculation tasks for computational efficiency, without a significant decrease in accuracy. For fast evaluation of the Coulomb and exchange integrals, a RIJCOX^15,71^ algorithm, which significantly accelerates the calculations and maintains high numerical precision, was used. The final energies were refined at the ωB97X-D4/def2-QZVPP^69^ level of theory to ensure the highest possible accuracy for the computed binding energies and act as a final reference point for the DFT-calculated binding energies. Single point energies (SPE) of unoptimized dimers were also calculated using the aforementioned methods without the ZPE and thermal corrections but with the BSSE correction to provide a direct comparison of the energy differences and act as a direct validation of the SMD-obtained energies. This comprehensive approach ensured that the energy differences were not artifacts of the optimization process. An increased grid (defgrid3) was used to reduce the numerical noise and increase the accuracy of the results. Through this approach, a robust comparison across different levels of theory was possible. The binding energies (Δ*E*) were calculated using the supramolecular approach:
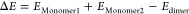
where *E*_monomer1_ and *E*_monomer2_ are the energies of individual monomers (optimized with ZPE, thermal and BSSE corrections or unoptimized only with BSSE correction) and *E*_dimer_ is the energy of the dimer. The same method of calculating Δ*E* was applied to both optimized geometries and unoptimized ones, e.g., directly taken from MD simulations with no additional steps. The starting coordinates and optimized coordinates for both R6G and anthracene dimers are provided in the Supporting Information.

### Steered Molecular Dynamics

SMD systems were prepared in a slightly modified way compared to the one presented before.^20^ Specifically, instead of continuing from an existing stable dimer, the most stable and representative dimers were reconstructed from scratch. The selected dimer was solvated with TIP3P^72^ and neutralized using NaCl, with water box padding set to 15 Å. The cutoff for the van der Waals interactions was set to 12 Å, while the electrostatic interactions were evaluated using the PME method. The obtained rectangular periodic simulation cell size was 44 Å × 44 Å × 45 Å with approximately 6800 atoms for R6G simulations and 41 Å × 35 Å × 38 Å and around 4500 atoms for the anthracene simulations. The differences in atom numbers between systems arose from the fact that each dimer had a slightly different conformation and size, which, in turn, affected the final box size. The system minimization was performed in the same two-step manner as that described in the MD section. Throughout the minimization process, all dimer atoms were constrained and fixed in place to prevent them from being pulled apart by the conjugate gradient and line search algorithm. The above ensured that each of the used dimers is unique and has a slightly different conformation than the others. To estimate the binding energy of the dimers using force field methods, an identical approach as presented in our earlier work was used,^20^ namely, a constant velocity (0.01 Å/ps) SMD pulling with a harmonic force constant of 4 kcal/(molÅ), equivalent to 278 pN/Å and an integration step of 1 fs, total SMD trajectory length was 2 ns. At lower pulling velocities, the simulation time scale became inefficient, whereas altering the force constant introduced significant noise into the trajectories, which dominated over the local unbinding potential. Furthermore, high velocity and spring constant values could lead to structural alterations of the pulled molecule. However, the RMSD data (Figures S2−S5) indicate that the structures were well preserved throughout the simulation and comparable to MD simulations.

Additionally, to assess the method’s precision, the SMD simulations for DFT-optimized anthracene and R6G dimer 1 were repeated 10 times. Given that the obtained values did not differ significantly in the case of 10 and 4 repetitions, i.e., 18.44 ± 2.46 kcal/mol for the R6G dimer from 10 repetitions vs 18.98 ± 2.05 kcal/mol from 4 repetitions and 7.36 ± 1.66 kcal/mol vs 7.23 ± 1.92 kcal/mol for the anthracene dimer, the remaining SMD simulations were repeated 4 times from the same starting point. To minimize the noise arising from the friction between the pulled R6G and the other dye molecule, one of the dimer components was fixed, and the other was pulled away in the direction perpendicular to the aromatic planes by applying the external force to all heavy atoms of that plane. To further minimize the noise, a constant temperature control was disabled to ensure that the disturbance caused by the molecular movement was minimal. Despite the temperature control being switched off, the temperature remained close to the set value of 300 K and fluctuated from around 296 to 302 K. The force and displacement plots as a function of simulation time combined with visual analysis were used to calculate the binding energies. The energy calculation method has been previously used for estimating the desorption energies of proteins with success,^32,34,73,74^ and it is described in the Supporting Information.

All MD and SMD simulations were performed using NAMD3 CUDA^75^ version with CHARMM36^76^ FF, and VMD^77^ was used to visualize and analyze the simulations. A sample SMD trajectory is provided in the Supporting Information.

## Results and Discussion

**Figure 1:**
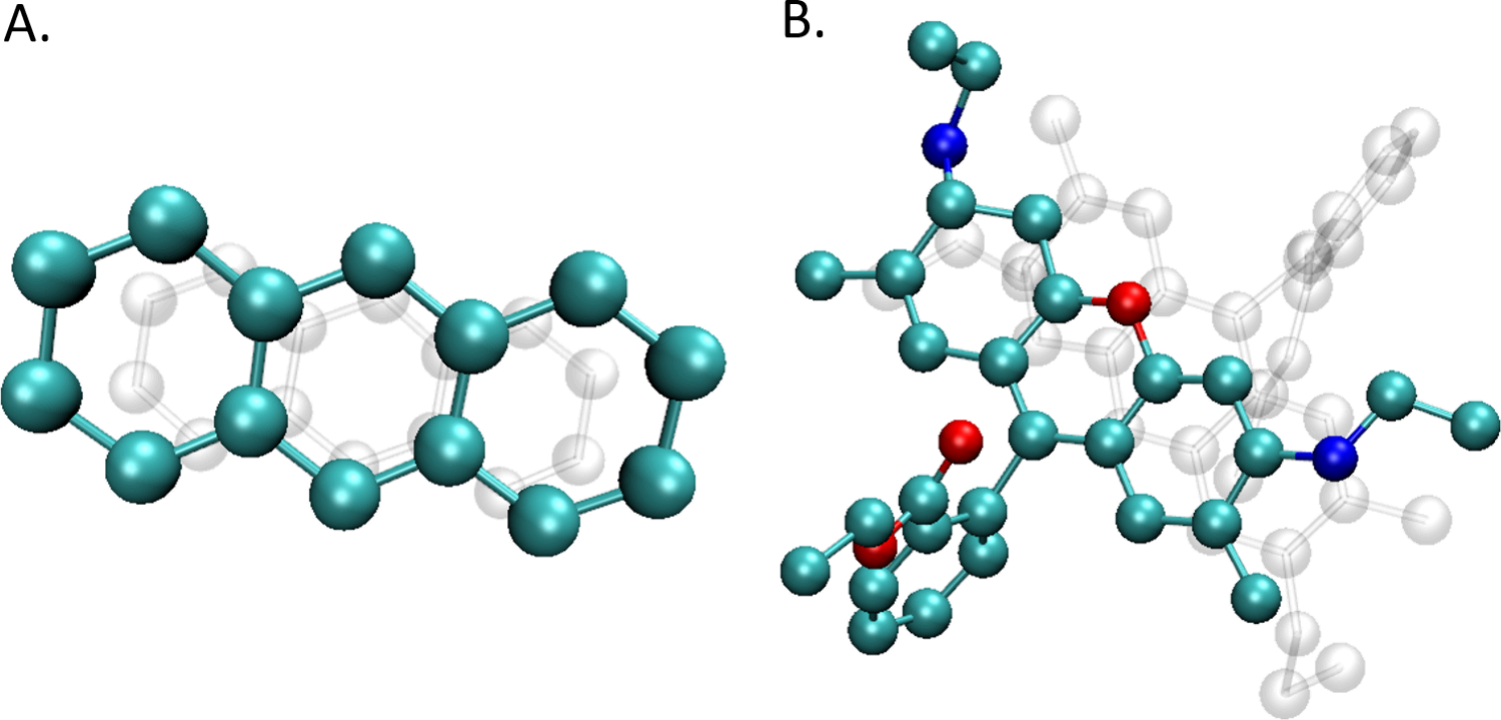
Starting dimer structures: (A) anthracene dimer and (B) R6G dimer. Structures are shown by ball and stick representation, colored by name: C, O, and N are shown in cyan, red, and blue, respectively, while hydrogens are omitted for clarity. Shadow (depth cueing) is used to distinguish separate monomers in the dimer structure.

As mentioned in the Methods section, to obtain a set of four independent dimers for both anthracene and R6G, a classical MD simulation was performed using CHARMM36 FF. Each 100 ns long simulation (without any additional constraints) generated distinct molecular conformations, providing a diverse set of dimer geometries. Such an approach ensured that the chosen dimers were not biased toward a particular conformation, thereby providing a robust starting point for subsequent DFT calculations and SMD simulations. Exemplar structures of anthracene and R6G dimers are shown in Figure 1.

### Density Functional Theory

A range of DFT calculations were performed to gain full insight into the magnitude of the binding energies of the dimers studied. These calculations aimed to provide a valid comparison with SMD simulations and ensure that the energy differences observed are intrinsic to the molecular interactions rather than artifacts of the optimization process. Initially, the electronic energies of each structure were calculated at multiple levels of theory, including generalized gradient approximation (GGA), hybrid GGA, hybrid-meta-GGA, and range-separated hybrid functionals. The specific functionals used were BP86, B3LYP, M06-2X, and ωB97X-D. This range of functionals allowed for a detailed comparison of how each level of theory captures electron correlation and exchange interactions, particularly the nonlocal components of HF exchange. Next, the structures were optimized at each level of theory to account for quantum mechanical effects such as Pauli repulsion, which are not explicitly defined in MD force fields. This step was crucial because MD force fields might fail to account for detailed electron–electron repulsions as they lack explicit terms to describe the interactions. This can result in potential overbinding of molecules. By optimizing the structures, we ensured that the geometries obtained were near the minimum on the potential energy surface for each dimer. For the DFT part, the main part of the analysis was centered around optimized structures as typically done in the field; however, calculations for the unoptimized geometries were also added. Although such calculations are uncommon and often lack significance due to the abundance of unoptimized structures, in this specific instance, they served as a valid basis for comparison with the SMD simulations.

#### 3.1.1 Anthracene

**Figure 2:**
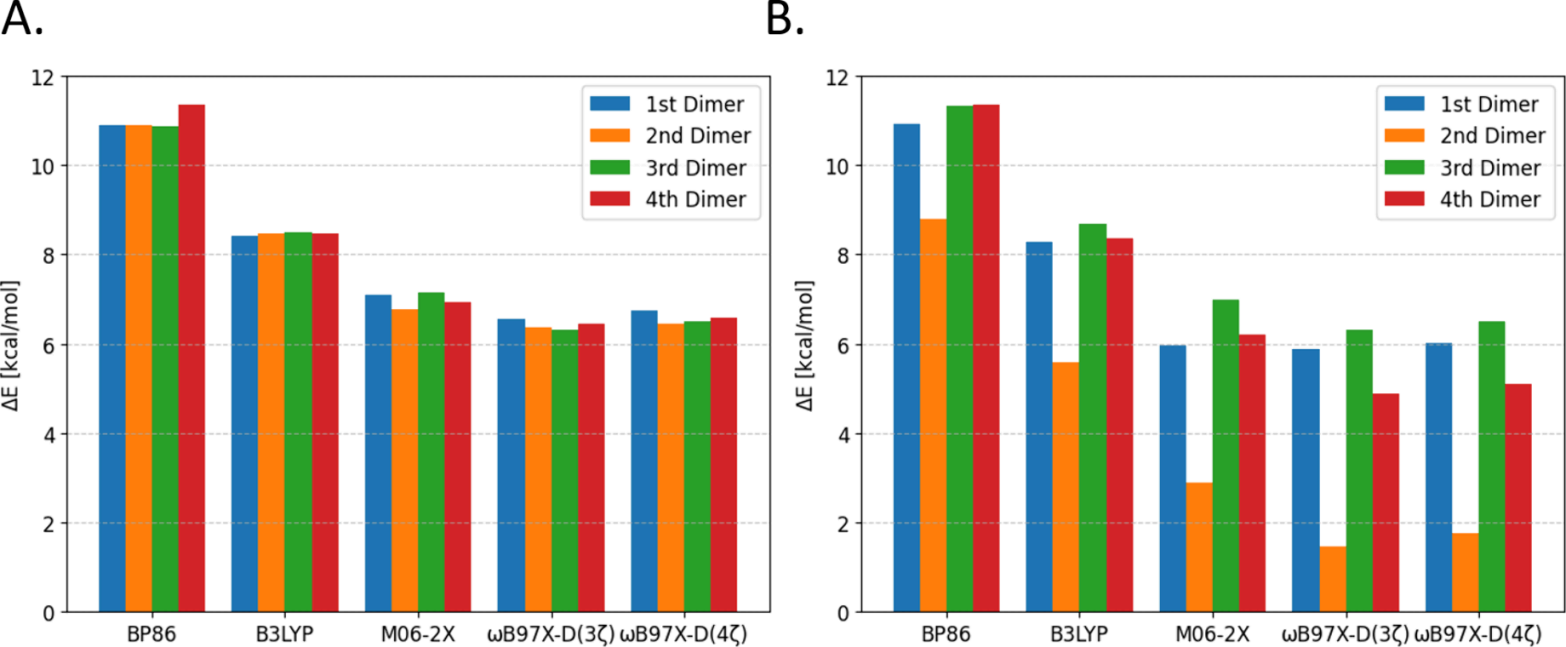
Binding energies of anthracene dimers at different levels of theory: (A) optimized structures; (B) unoptimized structures.

**Figure 3:**
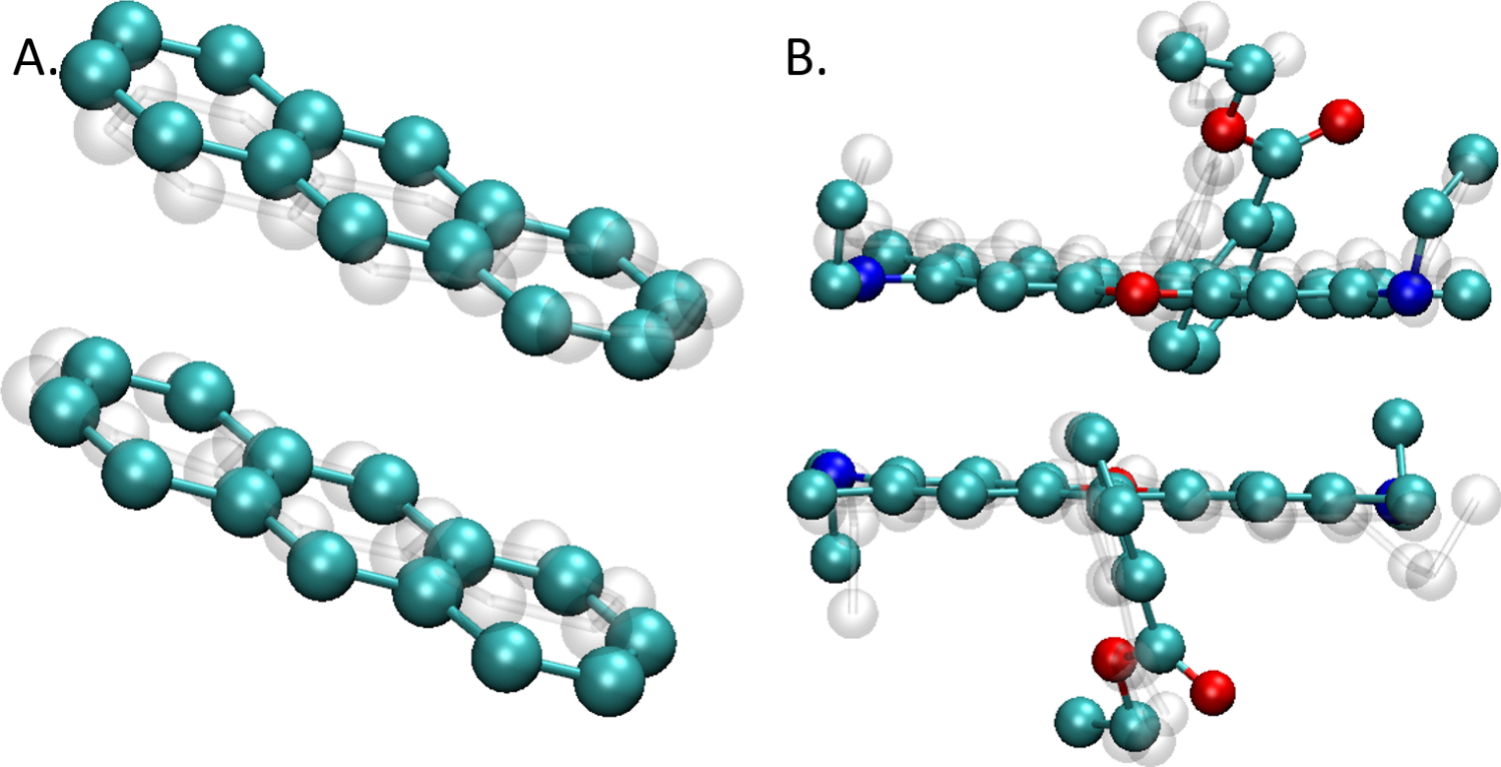
Overlap of optimized (colored, opaque) at ωB97X-D4/def2-TZVPP and starting (shadow/ghost) dimer structures. (A) Anthracene dimer and (B) R6G dimer. The coloring scheme is the same as that in Figure 1.

Binding energies obtained for optimized and unoptimized anthracene dimers are shown in Figure 2. For the optimized structures (Figure 2A), the energies of four independent dimers are virtually identical among all of the functionals used, suggesting that the obtained geometries are likely near the same local minimum. The most significant difference in the binding energies are observed for the transition from BP86 to a B3LYP global hybrid, with Δ*E* decreasing on average from 11 to 8.5 kcal/mol. BP86 belongs to the family of GGA functionals, and while it does offer some improvement over LDA functionals, it does not involve an exact (nonlocal) component of HF exchange but rather accounts for electron repulsion by considering density gradients. As a result, it fails to capture the electron repulsion fully, hence overbinding the molecules, highlighting a common limitation of GGA functionals.^78,79^ This is well reflected by a significantly higher Δ*E* at BP86-D4/def2-TZVPP when compared with other functionals, arising from the fact that the rest of the presented methods are hybrid and include a part of the HF exchange. The energy decreases further when moving from B3LYP (Δ*E* = 8.5 kcal/mol) to M06-2X (Δ*E* = 7 kcal/mol), which can be attributed to a high nonlocality of M06-2X, which includes 54% of HF exchange and offers a better treatment of dispersion interactions. The difference between M06-2X and range-separated hybrid ωB97X-D is relatively low, with Δ*E* decreasing from 7 to 6.4 kcal/mol, suggesting that there is not much improvement in the electron–electron correlations, thus indicating that once a certain level of HF exchange and nonlocal treatment is included, additional improvements in electron correlation might yield diminishing returns in the form of increased computational costs at a marginal improvement in accuracy. Therefore, careful consideration is required when selecting the appropriate functional; as in some cases, a more cost-effective global hybrid may yield results comparable to range-separated hybrids. A comparison of optimized vs unoptimized geometries is presented in Figure 3A.

As expected, despite the general similarity in decreasing energies with the increasing level of theory, the situation for the unoptimized dimers (Figure 2B) is less consistent with visible differences in Δ*E* between the particular structures, as each of the dimers has a different conformation. This further underscores the sensitivity of the binding energy to conformation, reflecting the realistic scenario where molecular flexibility and varied intermolecular interactions play a crucial role. The most interesting case is noted for dimer #2, where the decrease in the binding energy is the most significant with the increase of the functional capability to describe exchange-correlation energy. This discrepancy implies that the geometry of dimer #2 was relatively far from optimal; hence, DFT reported low Δ*E* (weak dimer binding). For the BP86, which has no HF exchange and has limited capabilities, the relatively high binding energy of 8.8 kcal/mol is a result of the overbinding, mentioned previously. The decrease in Δ*E* at higher levels of theory arises from the fact that electron repulsion starts to dominate; hence, the binding energy is very low and equal to 1.45 kcal/mol at ωB97X-D. This is the drawback of the proposed MD method, as force field methods do not include electrons explicitly; hence, estimation of the repulsive term is impossible. This can potentially result in an overestimated stability of certain dimers as is the case of anthracene dimer #2. As a result, the formed dimer will be stable (and indeed was) as observed during the MD trajectory, while the DFT calculations suggest that the Δ*E* is almost negligible.

#### 3.1.2 Rhodamine 6G

**Figure 4:**
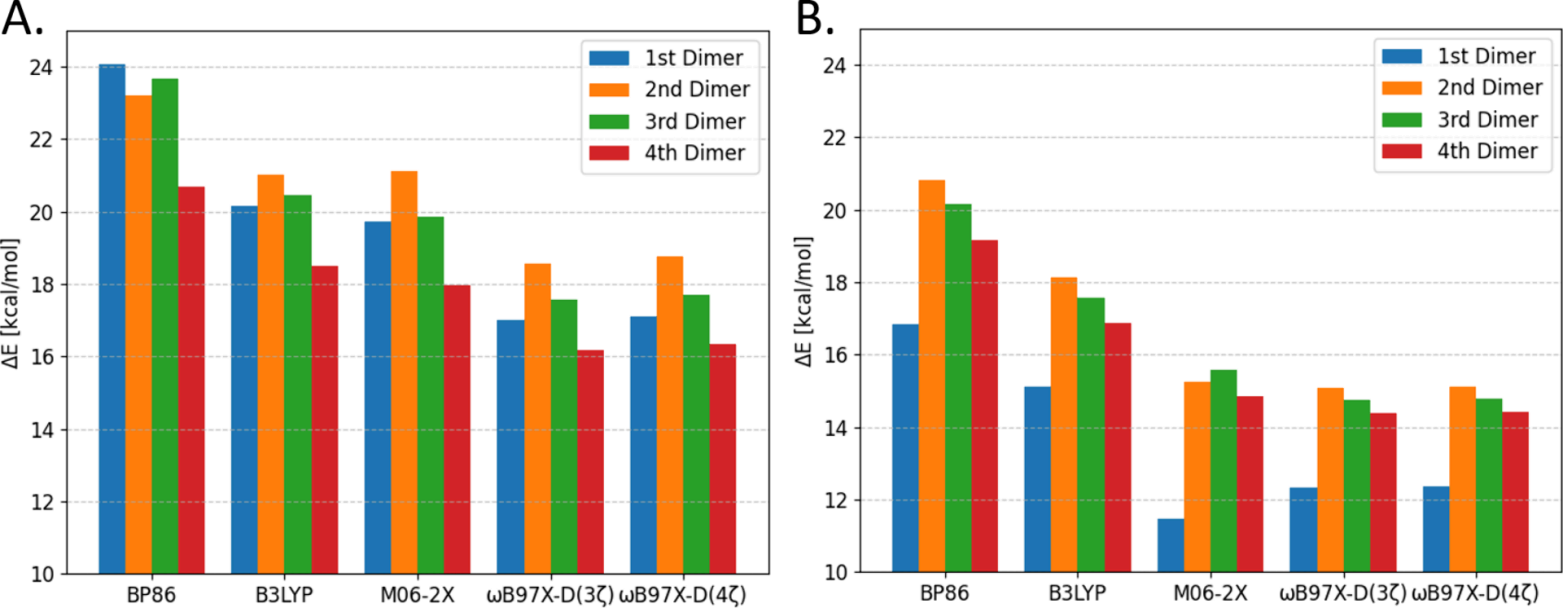
Binding energies of R6G dimers at different levels of theory: (A) optimized structures; (B) unoptimized structures.

Binding energies obtained for optimized and unoptimized R6G H-type dimers are listed in Figure 4. In general, the trends observed for the anthracene dimers were also observed in the case of R6G dimers. However, given that R6G is more complex than the anthracene molecule, with a greater number of atoms and numerous side chains, generated set of dimers represent a wider set of possible conformations than observed for anthracene. This variation may result in distinct local minima on the potential energy surface (PES).

H-Type R6G dimers are formed via van der Waals interactions and not by an explicit bond; hence, a precise estimation of dispersion forces and correct treatment of electron–electron interactions is vital. When examining the binding energies of optimized dimers (Figure 4A), it is again evident that more sophisticated functionals provide a better treatment of dispersion forces and electron–electron interactions. For example, the transition from the BP86 functional to B3LYP shows an average decrease in Δ*E* equal to 2.9 kcal/mol, indicating that the inclusion of exact HF exchange in B3LYP and other hybrid functionals leads to more accurate energy calculations. When looking at B3LYP and M06-2X, it is evident that the differences between the energies of optimized dimers are around only 0.5 kcal/mol, suggesting that the geometry does not change significantly between the two, which was indeed the case; however, caution must be taken when using M06-2X. Both B3LYP and M06-2X are global hybrid functionals that mix a portion of exact HF exchange with DFT exchange-correlation, providing a more accurate description of electron–electron interactions. Specifically, B3LYP includes around 20% HF exchange, while M06-2X includes 54%, which accounts for its slightly lower Δ*E* values due to a better dispersion treatment. Furthermore, the results from M06-2X dimer #1 seem to not follow the general trend, where the binding energy decreases with increasing levels of theory. This can be attributed to its heavy parametrization based on empirical data.^80^ Due to this high empiricism, this functional might be less predictive outside the types of systems and reactions that it was trained on, thus raising concerns about its generalizability to novel systems or conditions not represented in the training data.^81^ Furthermore, it seems that M06-2X tends to overestimate the binding energies and leads to other errors, such as overfitting, especially in cases, where a delicate balance of forces is critical.^82^ As a result, the predicted binding energies using M06-2X are closer to that of B3LYP, while the computational cost of M06-2X is comparable with ωB97X-D, due to its high numerical noise and a need for a finer DFT integration grid when performing the calculations.^83,84^ Lastly, a second steep decrease of around 2.3 kcal/mol is observed when transitioning from regular hybrid functionals to a range-separated ωB97X-D functional. Range-separated hybrids use different portions of HF exchange to treat long-range and short-range interactions, leading to an improved correlation treatment compared to global hybrids. This results in more precise binding energies, albeit at a significantly increased computational cost. The final refined energies for ωB97X-D4/def2-QZVPP, which involve a highly accurate and computationally demanding basis set, suggest that the estimated energies with triple-ζ basis sets were already close to the complete basis set limit for the system studied.

The results for the unoptimized structures shown in Figure 4B generally show similar trends. Moreover, dimer #1 seems to show similar traits to anthracene dimer #2, as it does not follow perfectly the trend of other dimers and its Δ*E* value is lower overall than other R6G dimers. The overall binding energies at each level of theory are lower for the unoptimized structures when compared with optimized structures, e.g., 17.11 kcal/mol for the optimized vs 12.34 kcal/mol for the unoptimized at ωB97X-D4/def2-QZVPP, which is mainly caused by the change in the position of all side groups. Furthermore, there are no steep decreases when transitioning from GGA to hybrid functionals and from hybrid to range-separated hybrid as in the case of optimized geometries. Here, the energy decreases continuously with the increasing complexity of the functional, which directly corresponds to the functional capabilities of accounting for exchange-correlation and describing electron repulsion, with BP86 being the simplest and fastest method of all and ωB97X-D being the most precise but also the most computationally demanding. Lastly, it is important to note that due to the complex structure of R6G and the flexibility of its side group, the DFT binding energies are significantly influenced by the position of these side groups, as that is the source of the largest difference between optimized and unoptimized structures as visualized on Figure 3B.

The generally consistent trends observed across both the anthracene and R6G dimers highlight the robustness of the computational methods used. For H-type dimers, which rely heavily on van der Waals interactions, the choice of functional is crucial. By including these detailed analyses, we emphasize the importance of selecting appropriate computational methods for accurately modeling molecular interactions, as heavily parametrized functionals, such as those from the Minnesota family, can lead to potential errors, such as overbinding. These findings are particularly relevant for systems where van der Waals forces dominate, and precise dispersion corrections are necessary to avoid this. As discussed below, additional care must be taken when estimating the binding energies when using the proposed method, as SMD tends to overbind dimers, leading to increased binding energies.

### Steered Molecular Dynamics

**Figure 5:**
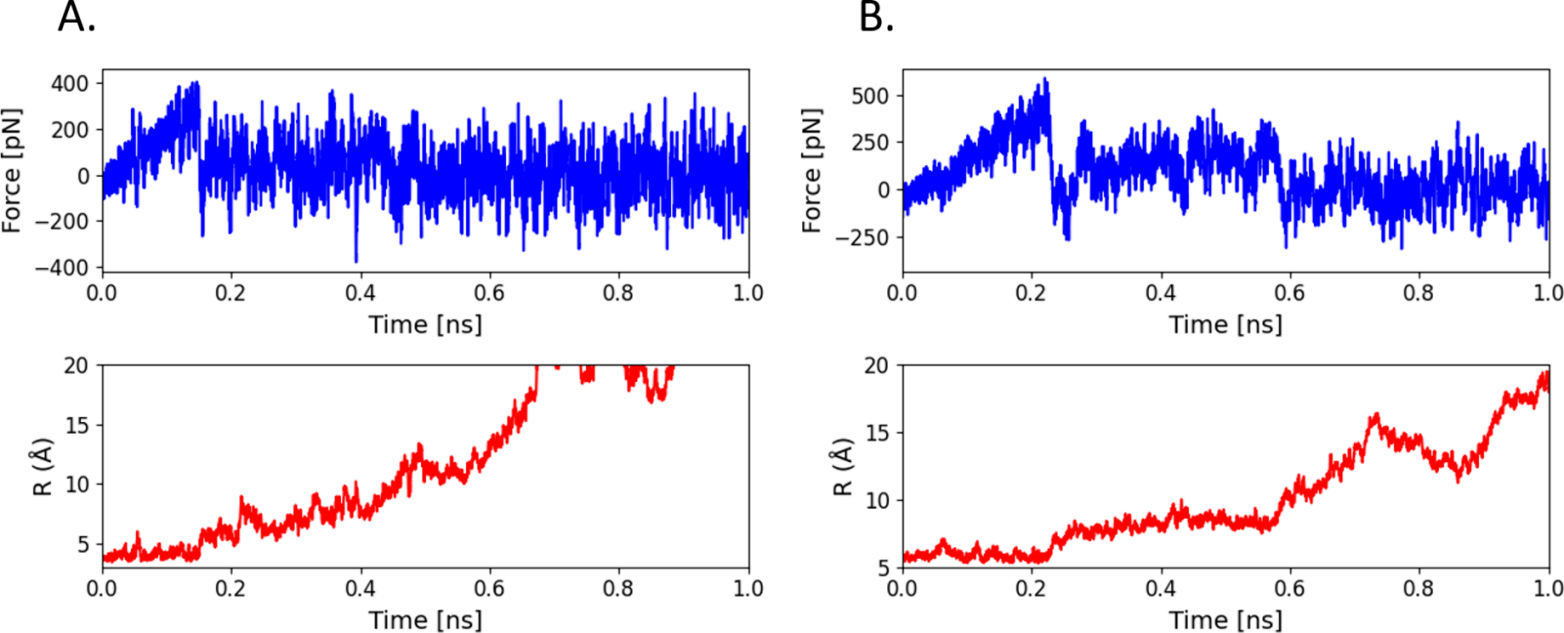
Exemplar SMD plots for the unoptimized geometries: (A) anthracene; (B) R6G. Top plots in blue represent the force change as a function of simulation time, while the bottom plots in red are the displacement as a function of simulation time measured between centers of masses of two monomers. As the dimer is fully dissociated at *R* = 10 Å, the displacement plots are created to enhance the interactions before that point.

To have a valid comparison of DFT-calculated binding energies with those obtained using SMD, we performed simulations for both optimized and unoptimized geometries. The main focus of the analysis was the unoptimized geometries as this is the typical approach for the SMD simulations. Nonetheless, to ensure a valid comparison with DFT calculations, SMD simulations were also performed on optimized structures (at the ωB97X-D4/def2-TZVPP level). This allowed us to assess whether force field methods can distinguish between different geometries successfully. In both cases, one of the dimer components was fully constrained by fixing the coordinates of all atoms (including hydrogens), while the other was pulled away at a constant velocity. To ensure that all bond-like interactions (including π-stacking) are broken simultaneously, the target molecule was pulled perpendicular to the aromatic planes with the force being applied to all heavy atoms of those planes. By combining visual analysis with force and displacement plots, dimer binding energies were estimated using methods from our previous work,^20,37^ as fully described in the Supporting Information. Exemplar SMD plots are shown in Figure 5. In all cases, dimer dissociation was a multistep process, typically involving two transitions before full dissociation. Namely, for R6G (Figure 5B), the first event was at around 0.23 ns and the second one was around 0.60 ns while; in a case of anthracene, they appeared earlier at 0.15 and 0.45 ns (Figure 5A). In all cases, the dimer was fully dissociated at 10 Å separation; therefore, any interactions past that mark are not taken into consideration. Furthermore, to obtain precise values of binding energies, any event that results in breaking of a bond-like interaction and subsequent creation of a new one must be excluded from the analysis. Finally, it is important to address the potential effect of the hydrodynamic drag in SMD simulations. As the target molecule is pulled through the aqueous environment, the spontaneous formation and breaking of hydrogen bonds reproduce a phenomenon that might correspond to friction as understood in macroscopic terms. Due to the short simulation time and small separation distance (the full dissociation happens at 10 Å distance), the magnitude of the resulting drag force is too small to be quantifiable, and it is already included in the SMD force reported by the software.

#### 3.2.1 Anthracene

The obtained results from SMD simulations for anthracene dimers are given in Table 1.

**Table 1:** Binding Energies of the Anthracene Dimers^a^

	dimer #1		dimer #2		dimer #3		dimer #4	
method	unoptimized	optimized	unoptimized	optimized	unoptimized	optimized	unoptimized	optimized
BP86-D4/(3ζ)	10.92	10.91	8.79	10.88	11.34	10.86	11.34	11.35
B3LYP-D4/(3ζ)	8.27	8.42	5.60	8.46	8.70	8.51	8.36	8.48
M06-2X-D3/(3ζ)	5.97	7.10	2.89	6.78	7.00	7.16	6.21	6.94
ωB97X-D4/(3ζ)	5.88	6.54	1.48	6.36	6.30	6.32	4.88	6.44
ωB97X-D4/(4ζ)	6.03	6.75	1.77	6.44	6.52	6.50	5.11	6.57
SMD	5.98 ± 1.49	7.36 ± 1.66	5.96 ± 1.09	7.93 ± 1.57	6.04 ± 1.14	7.99 ± 1.37	6.04 ± 1.35	7.41 ± 1.59

a All values are provided in kcal/mol, while errors for the SMD are taken to be equal to three standard deviations plus an estimated error of 0.58 kcal/mol arising from reading off the force values from the SMD plots.

When looking at the results for the unoptimized dimers, it is evident that, in the case of anthracene dimer, SMD is capable of predicting comparable binding energies of a dimer at a precision to that of hybrid DFT, e.g., 5.98 ± 1.49 kcal/mol from SMD vs 5.97 kcal/mol using M06-2X for dimer #1. The obtained Δ*E* values seem to be more accurate than those obtained using pure DFT with a BP86 functional, which contains no HF exchange, thus having limited capabilities in describing the exchange-correlation energy. Nonetheless, the SMD approach is not ideal, since due to the lack of explicit terms to account for electron interactions in CHARMM36 FF and lack of explicit evaluation of the hydrodynamic drag, the method tends to overbind dimers in some cases, as observed for dimers #2 (evident effect) and #4 (relatively minor effect). In the first case, as already found in DFT calculations, the dimer was virtually unstable with almost negligible binding energy, while the SMD obtained a value indicating that the dimer was stable. For dimer #4, no significant differences were observed in the SMD trajectories. Both visual inspection of the trajectory via VMD^77^ and COM plot analysis confirmed the stability of both of those dimers in MD. As a result of this overbinding, there were no statistically significant differences between all four dimer binding energies obtained from SMD results, while DFT indicated notably different Δ*E* for each of the dimers.

It is important to highlight that performing SMD on DFT-optimized structures is not a standard practice. Due to the dynamic nature of MD simulations, where the system evolves continuously over time, molecules often do not have sufficient time to fully relax into a local minimum. However, in this study, SMD simulations were applied to the optimized dimer geometries to validate the DFT results for these structures. The results demonstrate that after geometry optimization, all dimers had very similar geometries, as indicated by almost identical binding energies. As observed with unoptimized dimers, SMD successfully predicted binding energies comparable to hybrid DFT, with values such as 7.23 ± 1.92 kcal/mol from SMD vs 7.10 kcal/mol using M06-2X metaGGA functional. Furthermore, the overbinding effect previously observed in the case of dimer 2 and dimer 4 is prominent here through all dimers, with the predicted energies averaging around 1 kcal/mol higher than those predicted using a range-separated hybrid functional with a highly accurate 4ζ basis. In general, the SMD-observed energy difference between starting and optimized geometries was around 21%, while the DFT-observed difference was around 11%, excluding the outliers. Nevertheless, this study, and in particular the data listed in Table 1, demonstrates that SMD reliably predicts binding energies of simple anthracene dimers, yielding results consistent with hybrid DFT. Therefore, we can anticipate that this method can be applied to more complex polycyclic aromatic hydrocarbons (PAH) dimers; however, it should be used with caution.

#### 3.2.2 Rhodamine 6G

To further validate the method and gain more insight into the accuracy and capabilities of SMD in predicting binding energies, an analogous analysis was carried out on the R6G H-type dimer. The results for the R6G dimer are shown in Table 2.

**Table 2:** Binding Energies of the R6G Dimers^a^

	dimer #1		dimer #2		dimer #3		dimer #4	
method	unoptimized	optimized	unoptimized	optimized	unoptimized	optimized	unoptimized	optimized
BP86-D4/(3ζ)	16.84	24.09	20.81	23.20	20.15	23.66	19.18	20.69
B3LYP-D4/(3ζ)	15.13	20.15	18.12	21.01	17.56	20.46	16.87	18.49
M06-2X-D3/(3ζ)	11.45	19.74	15.24	21.13	15.59	19.87	14.86	17.98
ωB97X-D4/(3ζ)	12.35	17.00	15.09	18.58	14.75	17.57	14.40	16.19
ωB97X-D4/(4ζ)	12.35	17.11	15.13	18.75	14.79	17.71	14.44	16.34
SMD	10.46 ± 1.89	18.44 ± 2.46	14.76 ± 1.81	19.43 ± 2.38	15.17 ± 1.23	19.78 ± 1.89	15.05 ± 2.08	17.89 ± 2.59

a All values are provided in kcal/mol, while errors for the SMD are taken to be equal to three standard deviations plus an estimated error of 0.58 kcal/mol arising from reading off the force values from the SMD plots.

For the case of unoptimized structures, a similar trend was observed as with the anthracene dimer. One of the selected dimers (dimer 1) was found to be less stable than the other three, which aligns with the DFT calculations. However, the difference was not as significant as that found in the case of anthracene dimer #2. In general, both SMD and DFT showed comparable capabilities in detecting the dimers in less stable configurations; however, this was more prominent in DFT and slightly less noticeable in SMD simulations, as expected. These results demonstrate that the proposed SMD approach allows the differentiation of variations in dimer geometries as the obtained Δ*E* values are different for unoptimized and optimized dimer geometries, e.g., 10.46 ± 1.89 kcal/mol vs 18.98 ± 2.05 1.89 for dimer #1. Observation of this difference was not surpris**i**ng; as after optimization, both monomers were oriented more favorably than in MD-generated structures, hence the higher binding energy. The differences between the unoptimized and optimized geometries were 27% on average; however, in the case of dimer #1 it was over 44%. The main reason for this difference is the presence of the side groups in R6G which are very flexible, and thus, their orientation significantly affects the final binding energy. Furthermore, the feature of overbinding observed in anthracene dimers is also prominent here, with typically obtained Δ*E* being on average 1.5–2 kcal/mol higher than those obtained by using ωB97X-D4/(4ζ). It is worth noting that the weaknesses of M06-2X, particularly its tendency for overbinding, are more pronounced here with the functional reporting energies closer to B3LYP while containing a notably higher fraction of HF exchange (20% in B3LYP vs 54% in M06-2X) and significantly increased computational costs approaching those of ωB97X-D. This further suggests that the use of highly parametrized functionals, such as Minnesota functionals, which are often used for systems stabilized by vdW interactions, might potentially lead to overfitting.

Additionally, it is worth mentioning that our previous results^20^ predicted slightly different binding energies of dimers to the values reported here, with SMD-predicted Δ*E* for R6G being equal to 8.52 ± 2.80 and 13.45 ± 3.18 kcal/mol using DFT and 10.23 ± 1.36 kcal/mol with 9.41 ± 0.64 for anthracene, respectively. Although the current results are slightly different, it is important to note that previously reported results were obtained for unoptimized structures by using a single DFT functional with no corrections applied (such as BSSE or vibrational corrections). Furthermore, the impact of the outliers and the MD overbinding feature was not considered either. Therefore, those earlier findings should be considered preliminary, serving as an introduction to the method, while the current work should be perceived as a full validation of the method. Despite these differences, good agreement between the two methods is evident in both cases.

## Conclusions

In this work, we reported a DFT validation of an earlier proposed SMD method of estimating the binding energies of aromatic dimers. As obtaining experimental binding energy values is notoriously difficult due to the nature of dimerization, we employed two computational methods: force field-based SMD simulations, which are extremely fast for aromatic molecules, but still report accurate binding energy values, and much more computationally demanding DFT calculations using functionals from various rungs of Jacob’s ladder. Furthermore, by using independent dimers from multiple MD simulation runs, we showed that multiple monomer orientations in a dimer are possible, which have a notable impact on the final binding energy, which is successfully captured in both SMD and DFT calculations. Since both types of calculations were performed on optimized and unoptimized dimer geometries, this allowed us to assess the sensitivity of the SMD method and further understand the impact of the dimer geometry on the binding energy values. This work shows a novel example of CHARMM36 FF validation and also shows that this particular FF can capture the noncovalent interactions of the aromatic system with high accuracy. The obtained average binding energies for optimized anthracene dimers obtained using ωB97X-D4/(4ζ) were 6.46 kcal/mol vs 7.64 ± 1.61 kcal/mol using SMD and 17.48 and 19.02 ± 2.22 kcal/mol for R6G H-type dimer. Furthermore, it was found that SMD can differentiate minor variations in the geometries, as the binding energies obtained for each dimer differed accordingly and consistently fell within the uncertainty range of the hybrid DFT-calculated values. In general, we have found that global hybrids such as B3LYP or HSE^85^ provide the best balance between accuracy and computational cost. The use of more complex functionals results in somewhat improved accuracy; however, due to increased computational costs, they might be too expensive for larger molecules. Caution is advised when using highly parametrized functionals like M06-2X, as they tend to overbind, exhibit limited accuracy outside their training data sets, and require a larger DFT integration grid, significantly increasing computational costs.

It is important to acknowledge the limitations of the proposed method. Due to the absence of explicit electron–electron interactions in MD force fields and the presence of unavoidable hydrodynamic drag in SMD simulations, the presented method tends to overestimate binding energies, often producing results closer to the global hybrids rather than range-separated hybrids. In some cases, such as anthracene dimer #2, SMD even predicted stable dimers when DFT indicated otherwise. However, despite these discrepancies, the overall trends and general agreement between SMD and DFT suggest that the method can be used effectively to estimate the binding energies of larger complexes with a significantly reduced computational cost compared to high-level DFT calculations.

This study further validates our previous work by comparing the accuracy of SMD with various DFT functionals, each containing a more complex description of electron interactions. Importantly, the proposed method is not intended to replace conventional DFT or *ab initio* calculations entirely but to serve as a complementary technique that can provide preliminary binding energy estimates at a significantly reduced computational cost. Furthermore, this work reinforces the quality of the CHARMM36 FF. Given that other popular force fields such as GROMOS^86,87^ and AMBER^88,89^ are based on similar parametrization principles, we expect that comparable results could be obtained using those FF as well.

## Data Availability

The custom TCL scripts for data analysis can be found at: https://github.com/DanielDoveiko/TCL-Scripts-.git. All data underpinning this publication required to reproduce the simulations are openly available from the University of Strathclyde KnowledgeBase at: 10.15129/74c3602c-f376-4e0f-baa4-e8107e727777. Any additional data needed will be shared upon reasonable request to the corresponding author.

## Supporting Information Available

MD exemplar (MPG)
R6G SMD (MPG)
(1) Anthracene dimer coordinates generated during the MD run and its optimized structure at ωB97X-D4/def2-TZVPP level of theory; (2) R6G dimer coordinates generated during the MD run and its optimized structure at ωB97X-D4/def2-TZVPP level of theory; (3) details of binding energy calculations based on SMD plots; (4) sample RMSD plots for Anthracene and R6G obtained during MD and SMD simulations and R6G structure overlaps (MD vs SMD structure); (5) exemplar MD trajectory; and (6) exemplar SMD trajectory (PDF)

## Supplementary Material

jp4c07867_si_001.mpg

jp4c07867_si_002.mpg
