# Supplementary material for: Binding Energy Calculations of Anthracene and Rhodamine 6G H-Type Dimers: A Comparative Study of DFT and SMD Methods
Source: J Phys Chem A. 2025 Feb 6;129(12):2946–57. doi: 10.1021/acs.jpca.4c07867 (PMC11956137; doi:10.1021/acs.jpca.4c07867)
Supplement: Supplementary file 3 — jp4c07867_si_003.pdf [file jp4c07867_si_003.pdf]

Binding Energy Calculations of Anthracene and Rhodamine 6G H-type Dimers: A Comparative Study of DFT and SMD Methods

Daniel Doveiko[1*], Karina Kubiak-Ossowska[2] and Yu Chen[1*]

[1] Photophysics Group, Department of Physics, University of Strathclyde, Scottish Universities Physics Alliance, Glasgow G4 0NG, U.K.

[2] Department of Physics/Archie-West HPC, University of Strathclyde, 107 Rottenrow, Glasgow G4 0NG, UK

[*] Authors to whom correspondence should be addressed: daniel.doveiko.2018@uni.strath.ac.uk and y.chen@strath.ac.uk

Supplementary information contains:

1) Anthracene dimer coordinates generated during the MD run and its optimized structure at ωB97X-D4/def2-TZVPP level of theory
2) R6G dimer coordinates generated during the MD run and its optimized structure at ωB97X-D4/def2-TZVPP level of theory
3) Details of binding energy calculations based on SMD plots
4) Sample RMSD plots for Anthracene and R6G obtained during MD and SMD simulations and R6G structure overlaps (MD vs SMD structure)

(1) Anthracene Coordinates

Starting Anthracene Coordinates:

48

1st Monomer

C	7.39700	-19.97900	15.61200
C	8.41800	-20.06700	14.61200
C	8.62000	-21.31500	13.91700
C	7.89600	-22.46200	14.34000
C	6.84300	-22.32400	15.27100

C	6.61900	-21.09300	15.92300
H	7.23800	-19.11400	16.22000
H	8.97300	-19.18100	14.36200
H	6.26700	-23.17800	15.62800
H	5.66700	-20.94400	16.45700
C	9.78800	-21.42500	13.18800
C	10.12600	-22.72600	12.66300
C	11.32200	-22.95700	11.96800
C	11.66400	-24.21700	11.45200
C	10.72200	-25.20900	11.60600
C	9.59100	-25.02100	12.35000
C	9.29300	-23.82900	12.95300
C	8.22200	-23.72200	13.83500
H	10.50000	-20.58900	12.94800
H	11.99000	-22.11600	11.74700
H	12.60300	-24.40000	10.85700
H	10.95300	-26.19200	11.26900
H	8.93900	-25.87900	12.72000
H	7.63700	-24.61600	14.00400

2nd Monomer

C	5.12300	-19.96900	12.60900
C	5.45400	-21.27600	12.23800
C	6.39000	-21.55100	11.17700
C	7.06600	-20.48200	10.64600
C	6.85800	-19.14100	11.09700
C	5.82500	-18.87000	12.04300
H	4.33300	-19.84900	13.32600
H	4.95300	-22.12500	12.60500
H	7.50000	-18.37900	10.66700
H	5.52100	-17.83600	12.37200
C	6.62600	-22.90900	10.79000
C	7.66300	-23.14400	9.90400

C 7.93500 -24.50400 9.54100

C 9.01800 -24.73900 8.60600

C 9.72500 -23.64600 8.11800

C 9.46900 -22.34500 8.52900

C 8.43500 -22.05800 9.39300

C 8.08000 -20.73500 9.68300

H 6.11500 -23.69100 11.16500

H 7.29500 -25.30500 9.96200

H 9.28600 -25.72600 8.34100

H 10.51300 -23.80200 7.43200

H 10.06700 -21.58500 8.02100

H 8.68100 -19.88400 9.31400

Optimized Anthracene Coordinates:

48

1st Monomer

C 7.33721749023396 -19.77758661601864 15.83762303362654

C 8.29461623885789 -19.92744487222737 14.88907355742802

C 8.59785386176790 -21.21769676550565 14.35012632025434

C 7.87380440595541 -22.35053076863185 14.83212278130054

C 6.87003076804648 -22.14940501435741 15.83248598262392

C 6.61017334937567 -20.90915105051224 16.31601384218709

H 7.11441284468924 -18.79601538171789 16.23756972301798

H 8.84598523008795 -19.07095666179791 14.51737267673881

H 6.32195417165134 -23.01201825733301 16.19439561105968

H 5.84820073455082 -20.76738028868701 17.07268314621776

C 9.57054903974556 -21.39723977613998 13.37048504650027

C 9.85801710433334 -22.65876562732611 12.85698520729862

C 10.85608278444754 -22.85800603605330 11.85060450942840

C 11.12147160419075 -24.09829354977811 11.37142448600853

C 10.40519281541720 -25.23140975080041 11.86081832254587

C 9.44772345067076 -25.08375843012641 12.80990833609505

C	9.13805643082462	-23.79231483806465	13.34337184424060
C	8.16008829456251	-23.61224244891574	14.31816783853223
H	10.11550756555603	-20.53531159500008	12.99852849777116
H	11.39337233852833	-21.99257305742947	11.47869073863169
H	11.87652344279036	-24.23840049360554	10.60729459043844
H	10.63252485042911	-26.21341135699289	11.46423738696694
H	8.89991809498686	-25.94155824044190	13.18356413493001
H	7.61057691938223	-24.47301641140831	14.68543183355141

2nd Monomer

C	5.35912168683254	-20.29317138924715	12.51634419452589
C	5.61798274718618	-21.53845705447972	12.04668494596912
C	6.61333762146636	-21.75034200557500	11.04011981568211
C	7.33774770088265	-20.62414392572925	10.54343316292687
C	7.03595166773696	-19.32720677526634	11.06813486932760
C	6.08063581988540	-19.16748582503381	12.01746930118923
H	4.60495693785361	-20.14341699797541	13.27954103341784
H	5.07732697740507	-22.39839019090787	12.42639976458327
H	7.58711477248184	-18.47495684114008	10.68680561653980
H	5.85852038336593	-18.18122973238002	12.40638711224594
C	6.89309282434758	-23.01700534503973	10.53510984366940
C	7.86244240561916	-23.20861457904865	9.55435189197349
C	8.15839601510322	-24.50437196801917	9.02463011347451
C	9.11388904236015	-24.66616458265500	8.07611219348048
C	9.84505787055013	-23.54181388663050	7.58727043923394
C	9.59148702159539	-22.29653787703463	8.06113820685464
C	8.59082220276332	-22.08307319319105	9.06200605711969
C	8.31207229506119	-20.81614275662570	9.56730647646349
H	6.34505320964152	-23.87348412175304	10.91502550023750
H	7.60421587248655	-25.35540776620508	9.40455146866413
H	9.33208691952768	-25.65204747051239	7.68430129594146
H	10.60521553340366	-23.69298078815094	6.83059244441855
H	10.14250695546135	-21.43920822987757	7.69123985328357

H 8.86449712189946 -19.96072074064680 9.19200235838187

(2) R6G Coordinates

Starting R6G Coordinates:

128

1st Monomer

C	-27.38500	31.10200	-7.01500
C	-28.06400	32.17400	-7.56700
C	-28.84900	31.92500	-8.78800
C	-29.60500	33.05400	-9.29700
H	-30.20100	32.74400	-10.07600
C	-29.59200	34.25400	-8.66700
N	-30.21500	35.28800	-9.37500
H	-30.24200	36.21000	-8.99900
C	-31.57300	35.00500	-9.91600
C	-32.52500	34.79100	-8.75000
H	-31.55400	34.11500	-10.57000
H	-31.87100	35.91900	-10.45000
H	-33.61000	34.93400	-8.96600
H	-32.40300	35.55900	-8.00200
H	-32.24300	33.88500	-8.28600
C	-28.79600	34.53500	-7.55100
C	-28.59200	35.87000	-6.94300
H	-28.96500	36.79500	-7.58700
H	-27.54700	36.08900	-6.66400
H	-29.09100	35.94400	-5.96000
C	-28.00400	33.47700	-7.05400
H	-27.46800	33.73900	-6.15800
O	-28.93800	30.65700	-9.34200
C	-28.41100	29.59500	-8.60600
C	-27.60100	29.70900	-7.43300

C	-26.96900	28.54500	-6.83000
H	-26.24900	28.63100	-5.96100
C	-27.19900	27.29300	-7.37500
C	-28.12700	27.14000	-8.55500
N	-28.85100	25.92300	-8.24300
C	-29.87500	25.47100	-9.18800
H	-29.24900	26.11100	-7.27400
H	-30.54800	26.31200	-9.52600
H	-30.48400	24.68500	-8.69600
C	-29.13200	24.91400	-10.39500
H	-28.40200	24.14400	-10.14700
H	-28.61000	25.77800	-11.00300
H	-29.81600	24.46500	-11.09600
C	-28.93000	28.31500	-9.00700
H	-29.27100	28.20400	-10.05400
C	-26.32700	26.17300	-6.77600
H	-25.63200	26.47000	-5.98500
H	-25.59800	25.64800	-7.46200
H	-26.94000	25.43400	-6.22800
C	-26.44000	31.39600	-5.84500
C	-26.80900	30.97200	-4.52700
C	-28.12600	30.40900	-4.03000
O	-29.10100	30.53000	-4.86400
O	-28.16200	29.84300	-2.97200
C	-30.28000	29.84800	-4.50400
C	-31.31400	30.13400	-5.60600
H	-30.58900	30.12700	-3.46700
H	-30.09800	28.70000	-4.55900
H	-31.45600	31.18600	-5.77900
H	-32.28200	29.71800	-5.37700
H	-31.04300	29.68200	-6.53200
C	-25.86700	31.19400	-3.50700

C	-24.62100	31.83600	-3.79100
H	-26.05900	30.85200	-2.50100
H	-23.92300	31.80400	-2.95900
C	-24.27500	32.18200	-5.12100
C	-25.16600	31.85900	-6.11800
H	-23.28300	32.58200	-5.24500
H	-24.91600	32.15100	-7.13000

2nd Monomer

C	-26.39200	30.73600	-11.95700
C	-26.27300	32.12800	-11.52500
C	-25.42300	32.31800	-10.37500
C	-25.16100	33.68000	-9.96000
H	-24.41700	33.84300	-9.21300
C	-25.70500	34.74100	-10.66200
N	-25.47900	36.01100	-10.17700
H	-26.34800	36.41000	-9.88100
C	-24.48300	36.40500	-9.11200
C	-23.07500	36.21200	-9.67300
H	-24.68700	35.86300	-8.19400
H	-24.58200	37.50300	-8.82900
H	-22.31900	36.43400	-8.88200
H	-22.90100	36.92200	-10.50500
H	-22.87600	35.27200	-10.11100
C	-26.57900	34.50900	-11.67900
C	-27.10800	35.70400	-12.37100
H	-26.33700	36.21100	-12.97600
H	-27.70300	36.36200	-11.60500
H	-27.85100	35.32500	-13.08600
C	-26.77100	33.21700	-12.15400
H	-27.32700	33.23400	-13.09200
O	-24.80000	31.32400	-9.83300
C	-25.01800	30.01700	-10.22900

C	-25.75600	29.62400	-11.34200
C	-25.92000	28.21800	-11.72600
H	-26.60000	27.77200	-12.40300
C	-25.12800	27.29700	-11.12300
C	-24.00200	27.71200	-10.16600
N	-23.70000	26.77400	-9.06800
C	-22.26000	26.80700	-8.65400
H	-24.23300	27.07700	-8.29000
H	-22.23600	26.09900	-7.80400
H	-21.93600	27.77300	-8.27300
C	-21.35100	26.34100	-9.78900
H	-21.61600	25.27200	-10.08400
H	-20.32800	26.37800	-9.37100
H	-21.23900	26.97400	-10.64700
C	-24.17800	29.03800	-9.56500
H	-23.37600	29.45800	-8.93100
C	-25.37300	25.88400	-11.56400
H	-26.25900	25.71200	-12.20000
H	-25.51100	25.19900	-10.68900
H	-24.48000	25.42000	-12.02300
C	-27.18700	30.52900	-13.23700
C	-26.53800	30.14800	-14.46300
C	-25.08300	30.04100	-14.62600
O	-24.41700	30.74500	-13.72400
O	-24.61600	29.29000	-15.49100
C	-23.03500	30.80800	-14.17400
C	-22.33200	31.55400	-13.02100
H	-22.83200	31.29200	-15.07300
H	-22.64000	29.78600	-14.34900
H	-22.24500	30.91000	-12.14000
H	-22.74600	32.52100	-12.84400
H	-21.26000	31.85300	-13.29500

C	-27.37600	29.93100	-15.61300
C	-28.79200	29.96900	-15.50900
H	-26.94800	29.63700	-16.56900
H	-29.40200	29.67700	-16.34600
C	-29.36000	30.33600	-14.29200
C	-28.59800	30.67400	-13.15500
H	-30.48000	30.36100	-14.20100
H	-29.08600	30.93500	-12.23200

Optimized R6G Coordinates

128

1st Monomer

C	-27.24762898379775	31.17979868082334	-6.98197853470857
C	-27.87775347116583	32.32150694027064	-7.48510578979407
C	-28.77586868802036	32.18161905177416	-8.56375611119203
C	-29.43601642142135	33.24836860853171	-9.12607529068119
H	-30.10700174643781	33.06586449725081	-9.95227506381528
C	-29.23197619098211	34.53332323489767	-8.60486702407774
N	-29.82916059694383	35.60955025406878	-9.13942709473905
H	-29.69566164174777	36.49497416489312	-8.68233054482987
C	-30.82807272924295	35.57117568208835	-10.19852131469643
C	-32.21561948652867	35.19167103794906	-9.68997540417552
H	-30.49687509389063	34.87983834105886	-10.97550404962396
H	-30.84895639317365	36.56474431871172	-10.64735931676052
H	-32.92553432677988	35.17267097131389	-10.51963551825435
H	-32.56417879756163	35.91814081016901	-8.95309932624673
H	-32.20706801837452	34.20454274181428	-9.22375783837069
C	-28.36150667196810	34.71285860137529	-7.46730590247905
C	-28.20903008904295	36.08003880495375	-6.86294228867488
H	-27.85004229762934	36.80711746996690	-7.59627917996415
H	-27.49917384784249	36.05417904538157	-6.03729103388493
H	-29.16651005892812	36.44665535415913	-6.48015549928379
C	-27.71712043460365	33.63257561760246	-6.95342984077304

H	-27.06322269053630	33.76667320946904	-6.10065560803096
O	-29.05264982839990	30.96343306378279	-9.08293196539941
C	-28.44117352252526	29.85125557574610	-8.61850715902141
C	-27.49539244458205	29.93756828844919	-7.57530400984054
C	-26.86476385937799	28.72203220716543	-7.18435055478872
H	-26.11088593681801	28.75606747847329	-6.40808995449206
C	-27.15958793396986	27.52719920529077	-7.75560415935179
C	-28.16072980169312	27.47609623922687	-8.79522488393729
N	-28.46897193727505	26.28951580663302	-9.33509047860525
C	-29.48999060538796	26.06206514656601	-10.35030371772083
H	-27.96581448930083	25.48224929671846	-9.01013740226763
H	-30.36844039960112	26.66469371164758	-10.10815629738835
H	-29.78786661108891	25.01682813810766	-10.26260463459140
C	-29.01082601369301	26.35322784776323	-11.76901781004544
H	-28.15960265704229	25.71944422343972	-12.02499926442281
H	-28.71144982107637	27.39557064975334	-11.88039290565599
H	-29.81605198213884	26.15494160066166	-12.47939890982673
C	-28.78690494858225	28.66033479778534	-9.21374719992900
H	-29.52363519804209	28.66409292780476	-10.00298928691663
C	-26.47190718497561	26.26681615772246	-7.31509496265760
H	-25.73976472815935	26.48960997892295	-6.54014034685164
H	-25.94890991218354	25.78514778989205	-8.14451151431792
H	-27.18806258176888	25.54502200581111	-6.91088172093926
C	-26.31566552678822	31.26596975679292	-5.82740171646697
C	-26.63745000830707	30.70612310223717	-4.58634149975182
C	-27.96319526940781	30.06841169791174	-4.31436485812605
O	-28.95914496505479	30.70471800748080	-4.92077548877024
O	-28.10638553367740	29.09564681689371	-3.60792559000817
C	-30.28236204247221	30.12959164639123	-4.80183792851431
C	-31.20912889763108	30.95558402001381	-5.66449959082145
H	-30.57254229698505	30.15249729909009	-3.75037275341772
H	-30.23484537379156	29.09028211931133	-5.13076739747229

H	-31.20523896706761	32.00098612544365	-5.34994745752946
H	-32.22607562008496	30.57004954901740	-5.57046827210144
H	-30.91603345715591	30.90192098135156	-6.71429186727431
C	-25.70773839747436	30.72629340098049	-3.55259915122528
C	-24.46609481913645	31.31744343407358	-3.73592587185813
H	-25.97186686759801	30.28419694162401	-2.59975087249125
H	-23.75090866321462	31.33843405384490	-2.92237893315914
C	-24.14682700680227	31.87872396647467	-4.96507893858298
C	-25.06641944288331	31.84990612645048	-6.00515954022394
H	-23.17725379823443	32.33702045604786	-5.12072696398062
H	-24.80568156769133	32.26988826622456	-6.96694283920947

2nd Monomer

C	-26.49409667876180	30.48735715951623	-11.52110348082592
C	-26.41301902036788	31.82117291785277	-11.18217215931212
C	-25.57684716512756	32.21802243174499	-10.09598045738488
C	-25.45859802929376	33.52145883266670	-9.69472584991112
H	-24.81204806957172	33.75447684407331	-8.86288852630965
C	-26.16640532955746	34.52413949441885	-10.37958514498819
N	-26.10489148470604	35.79622917779073	-9.99810271602733
H	-26.60037261463213	36.47866225332042	-10.54751598197260
C	-25.29073493841285	36.30598799594681	-8.89947180506194
C	-23.82325694602950	36.47307776679300	-9.27968366301418
H	-25.40234689282119	35.64064733924017	-8.04132471596029
H	-25.71757084291361	37.26778113854574	-8.61728236636649
H	-23.26812812729878	36.87824935571131	-8.43144241566575
H	-23.72320359015362	37.16206140868228	-10.12066790441693
H	-23.37196288021248	35.51943493896680	-9.55877225343157
C	-26.97169084220894	34.17697701355847	-11.53809901405388
C	-27.65249669955216	35.26669490043210	-12.31544956545584
H	-26.92325379412969	35.97494562127618	-12.71970031382719
H	-28.34769776847835	35.82926283817242	-11.68694009399681
H	-28.21333765962957	34.84203165100448	-13.14666015927046

C	-27.07673981892563	32.87820997371826	-11.89360455093860
H	-27.67195050598322	32.61823029749242	-12.75876042720561
O	-24.85469618164939	31.31014743295036	-9.41945377294320
C	-24.88256800161747	29.99927908001175	-9.76838118973733
C	-25.71088944269153	29.53597235431801	-10.79141745717855
C	-25.68782237905987	28.15413794506944	-11.05889123413823
H	-26.36564666639561	27.75366949517069	-11.80014881252974
C	-24.84436757156399	27.28593416576049	-10.40589158864963
C	-23.99142046733080	27.80413919195589	-9.39804948000407
N	-23.11552335283342	26.94578750261157	-8.67986388193397
C	-21.73755269978708	26.89746381435273	-9.21801354285910
H	-23.04823806781191	27.29272142758327	-7.73065441757888
H	-21.14626510787853	26.33274341407192	-8.49448289602378
H	-21.31437874285834	27.91071312601663	-9.26721635138881
C	-21.63493285187954	26.24529104548502	-10.58647322725249
H	-22.00825643567864	25.22038200534647	-10.56616992024376
H	-20.58770640803488	26.22519022593827	-10.89554286942793
H	-22.19273338773976	26.80423644788011	-11.34082845051456
C	-24.03710647289647	29.14946273282195	-9.07377989846415
H	-23.40262937302061	29.55174050418958	-8.29279142702757
C	-24.88443712728314	25.82137273516897	-10.75362769455808
H	-25.91177783743535	25.51414280065266	-10.95341183060115
H	-24.48046242855976	25.21283395179920	-9.94712730626843
H	-24.29553263014174	25.61878249274945	-11.65043583062236
C	-27.39455896752847	30.03486564037976	-12.61070144622530
C	-26.92641283777633	29.37234597009503	-13.75211767767390
C	-25.47055689045383	29.14311422621818	-14.00603410072774
O	-24.72141270225745	30.14239957707552	-13.55316487665924
O	-25.03358189860986	28.16289971065524	-14.56542012516356
C	-23.28551972722022	29.95515403940220	-13.58471215696525
C	-22.66071923125072	31.17368959568676	-12.94478937232473
H	-22.97556164822169	29.83018666058396	-14.62283911901953

H	-23.05332963524909	29.04027530094018	-13.03572287533042
H	-22.97545557292993	31.27366885482350	-11.90446956837191
H	-22.93625802269618	32.08090499033063	-13.48558819498953
H	-21.57386540680995	31.07452147263672	-12.96834403196045
C	-27.82566679061022	28.90594740559318	-14.70344485875803
C	-29.18872219448301	29.11175394864984	-14.54479529860243
H	-27.44392358414676	28.39183541501246	-15.57679557843774
H	-29.88148122114035	28.75262090680628	-15.29627834709664
C	-29.65603449922940	29.78080290326034	-13.42245178147722
C	-28.76417499804764	30.23609014850089	-12.46089015061815
H	-30.71831108574366	29.94652465166552	-13.28767530055132
H	-29.13464015689391	30.74135599528339	-11.57841424868539

(3) SMD Theoretical Contemplations

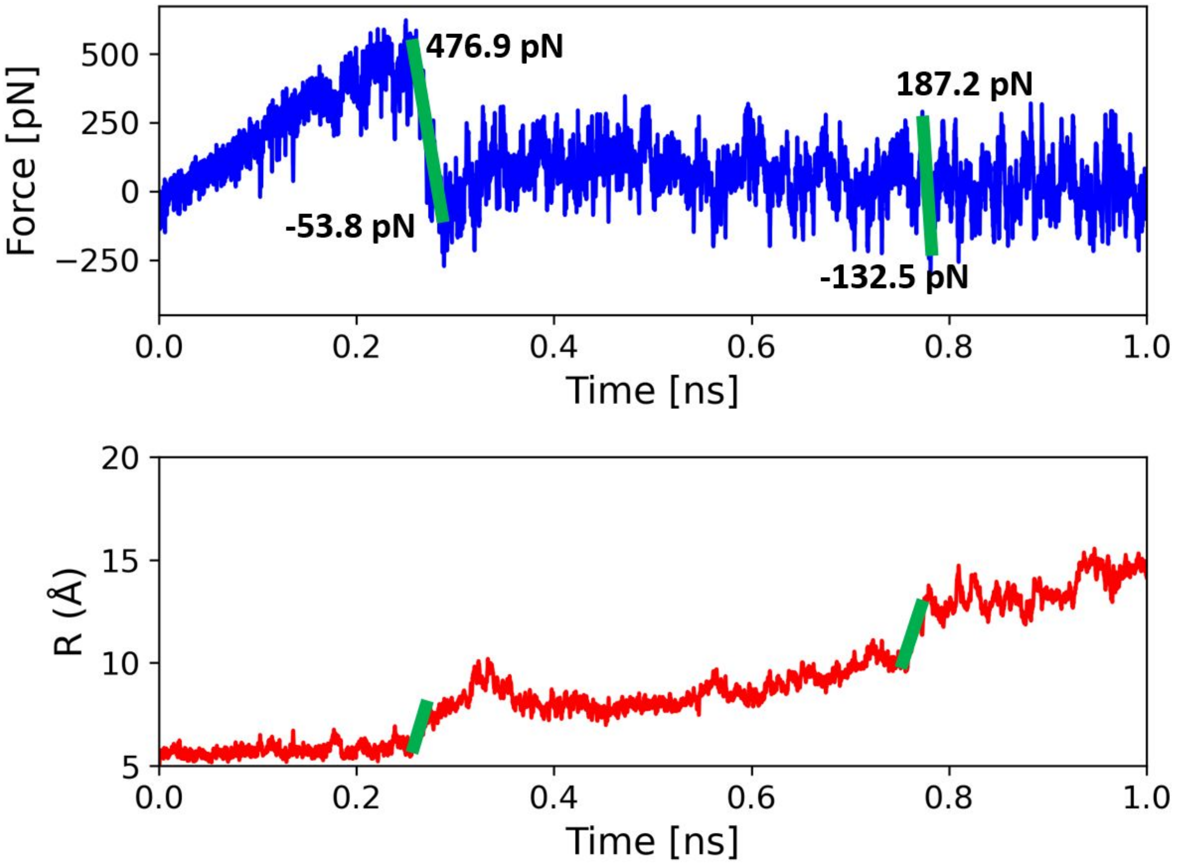

Figure S1 Exemplar SMD plot for optimized at ωB97X-D4/def2-TZVPP R6G dimer with the dimer dissociation steps marked.

To calculate the binding energy (dE) using the proposed SMD method, one needs to start by plotting the force and displacement as functions of simulation time. Next, using software capable of interactive plot analysis, it is necessary to identify the dissociation steps, characterized by sharp drops in the force plots and corresponding increases in the displacement plots, as indicated by the green bars in Fig. S1. The identified steps must then be confirmed through visual analysis of the trajectories, using software such as VMD or similar tools. After verification, the binding energy can be calculated using the equation provided below (Eq. 1). If the dissociation occurs in two steps (as in the presented case), the calculation (Eq. 1) must be performed for each step, and the final energy barrier is obtained as the sum of the two sub-terms. Here, the final energy barrier, dE, is calculated to be 18.69 kcal/mol. It is important to note that any dissociation steps involving the breaking of a bond-like interaction followed by the formation of a new one should be disregarded; hence, visual inspection of the trajectories is essential.

The formula is a simple potential energy of a spring formula but written in terms of the SMD measurable quantities:

$$dE = \left(F_0 + \frac{dF}{2}\right)\left(\frac{dF}{k}\right) \quad (1)$$

Assuming, that initially, our virtual spring is at rest, hence $F_0 = 0$. We get:

$$dE = \frac{dF}{2}\frac{dF}{k} \quad (2)$$

Knowing that:

$$dF = -kdx \tag{3}$$

This gives:

$$dE = \frac{(dF)^2}{2k} = \frac{k^2dx^2}{2k} = \frac{1}{2}kdx^2 \tag{4}$$

which is simply the potential energy of the spring formula.

(4) Root Mean Square Deviation (RMSD) plots

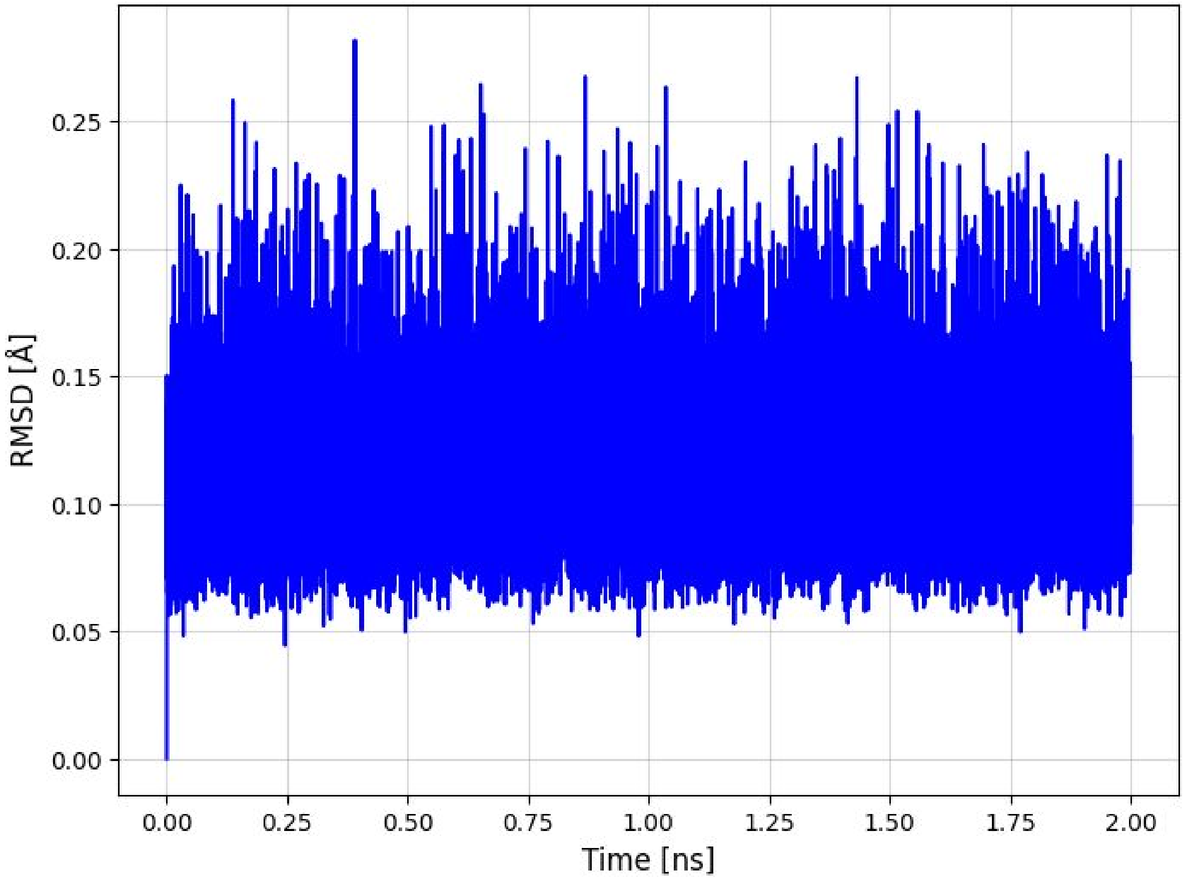

Figure S2 RMSD plot of pulled Anthracene molecule during the SMD simulation

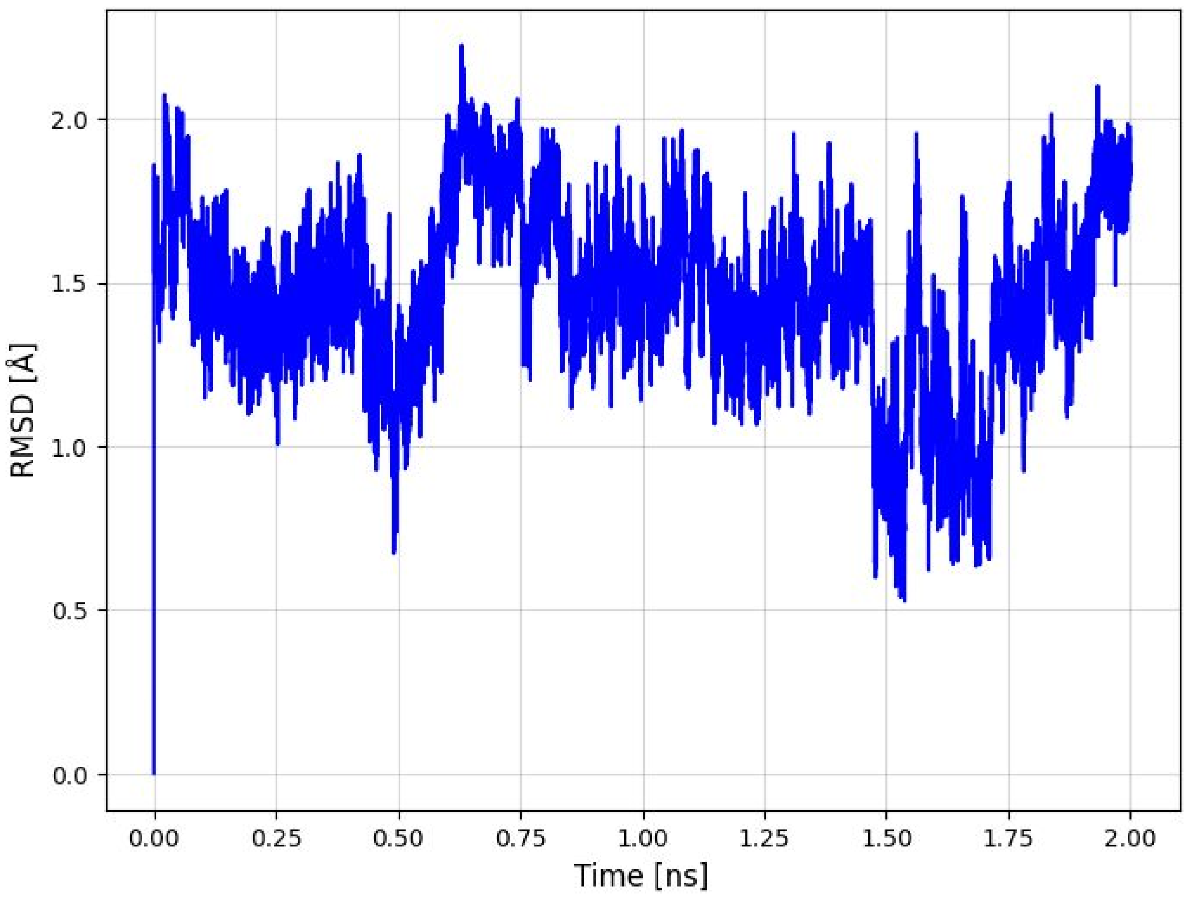

Figure S3 RMSD plot of pulled R6G molecule during the SMD simulation. Starting MD structure used as a reference structure for the plot.

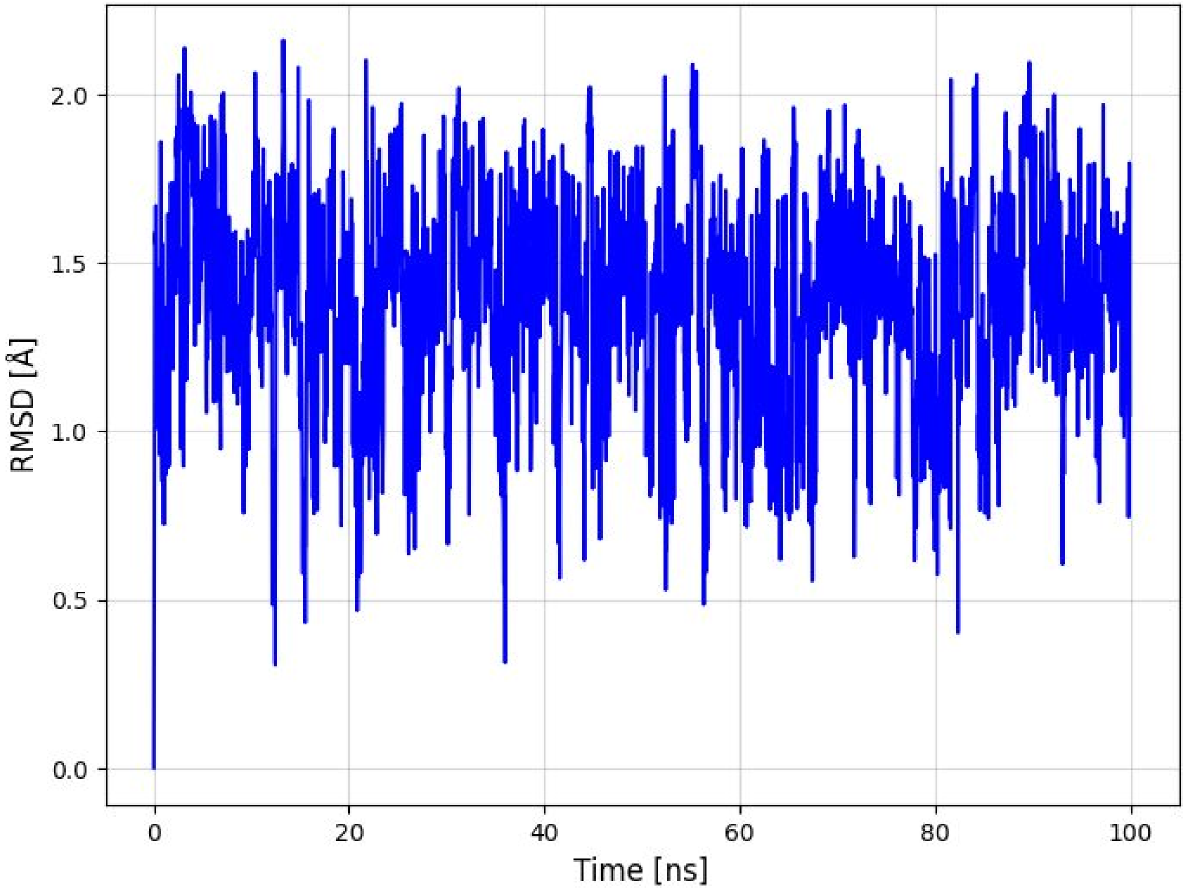

Figure S4 RMSD plot of pulled R6G molecule during the MD simulation. Note that the RMSD values during free diffusion are in the range of the values observed during the SMD simulation.

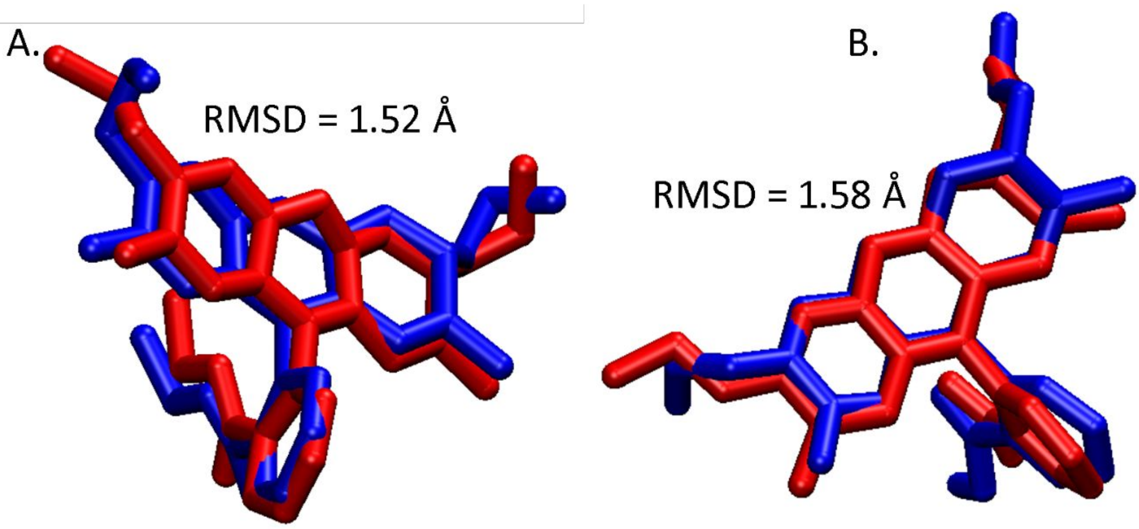

Figure S5 Overlaps of R6G structures during SMD (red) and MD (blue) trajectories.
